# Phylogenetic analysis of fungal ABC transporters

**DOI:** 10.1186/1471-2164-11-177

**Published:** 2010-03-16

**Authors:** Andriy Kovalchuk, Arnold JM Driessen

**Affiliations:** 1 Department of Microbiology, Groningen Biomolecular Sciences and Biotechnology Institute and Zernike Institute for Advanced Materials, University of Groningen, Kerklaan 30, 9751 NN HAREN, The Netherlands; 2 Kluyver Centre for Genomics of Industrial Fermentation, P.O. Box 5057, 2600 GA Delft, The Netherlands

## Abstract

**Background:**

The superfamily of ABC proteins is among the largest known in nature. Its members are mainly, but not exclusively, involved in the transport of a broad range of substrates across biological membranes. Many contribute to multidrug resistance in microbial pathogens and cancer cells. The diversity of ABC proteins in fungi is comparable with those in multicellular animals, but so far fungal ABC proteins have barely been studied.

**Results:**

We performed a phylogenetic analysis of the ABC proteins extracted from the genomes of 27 fungal species from 18 orders representing 5 fungal phyla thereby covering the most important groups. Our analysis demonstrated that some of the subfamilies of ABC proteins remained highly conserved in fungi, while others have undergone a remarkable group-specific diversification. Members of the various fungal phyla also differed significantly in the number of ABC proteins found in their genomes, which is especially reduced in the yeast *S. cerevisiae *and *S. pombe*.

**Conclusions:**

Data obtained during our analysis should contribute to a better understanding of the diversity of the fungal ABC proteins and provide important clues about their possible biological functions.

## Background

ATP-binding cassette (ABC) proteins constitute one of the largest protein families, with the number of known members exceeding 10,000 while expanding rapidly with each new genome sequence that becomes available [1]. ABC proteins are present in every living cell, ranging from archaea and bacteria to higher eukaryotes [2]. The majority of the ABC proteins characterized so far are involved in the ATP-dependent transport of a remarkably broad range of substrates across biological membranes. However, their role is not limited to active transport as some of them function as ion channels or receptors or are even involved in mRNA translation and ribosome biogenesis. The importance of ABC transporters is emphasized by the fact that they often contribute to multidrug resistance in microbial pathogens and tumor cells whereby they interfere with an effective treatment of infectious disease and cancer [3-5]. Mutations in many of the human ABC genes are linked to the hereditary diseases such as cystic fibrosis, adrenoleukodystrophy, disorders of cholesterol metabolism and other diseases [6,7].

**Figure 1:**
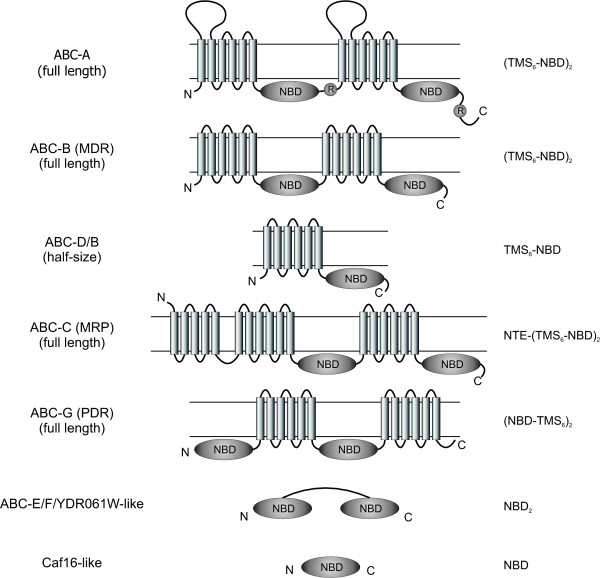
**Predicted topology and domain organization of different subfamilies of fungal ABC proteins**. NBD, nucleotide-binding domain; NTE, N-terminal extension; TMS, transmembrane segment. Modified after [14].

The structure of a prototypical ABC transporter includes four core domains, two nucleotide-binding folds (NBFs) and two transmembrane domains (TMDs) that may be expressed as separate polypeptide chains or, alternatively, as multidomain proteins. Two arrangements are common for eukaryotic ABC transporters. The functional unit is either composed from two "half-transporters", each containing its own TMD and NBF, or there is one large polypeptide chain that includes all four domains (Figure 1). ABC proteins not involved in membrane transport generally lack the TMDs. Within the NBFs, there are several conservative motifs that allow for an easy identification of ABC proteins within genome sequences. Those motifs include the so called Walker A and Walker B boxes [8], separated by ~120 amino acid residues, and an ABC signature motif situated between the two Walker boxes [9].

In the past, several classification schemes were proposed to accommodate the diversity of ABC proteins [2,6,7,10,11]. The Human Genome Organization (HUGO)-approved scheme was initially introduced to classify ABC proteins of vertebrates, however, nowadays it is also widely used for invertebrate and plant ABC protein [6,12,13]. According to this scheme, all eukaryotic ABC proteins are divided into eight major subfamilies, from A to H. An additional group I was recently proposed to include 'prokaryotic-type' ABC proteins in eukaryotic genomes [12]. This classification reflects the phylogenetic relationships between ABC proteins, allowing an assignment into structural classes based on sequence comparison of their NBF motifs. Here we have applied this scheme to fungal ABC proteins in order to facilitate their comparison with plant and animals, and to investigate their diversity and distribution.

Prior to the genomic era, the study of fungal ABC proteins was mainly performed with two model species, i.e., the budding yeast *Saccharomyces cerevisiae *and, to a lesser extent, fission yeast *Schizosaccharomyces pombe*. During the last two decades, this has led to a significant insight in the diversity and function of ABC transporters [14-17]. *S. cerevisiae *was also the first eukaryote whose genome was sequenced and for which a complete list of ABC proteins became available [18]. Yeast ABC transporters have quite diverse physiological functions, ranging from the secretion of the mating **a**-factor peptide, the biogenesis of iron-sulfur proteins to fatty acid metabolism and cell protection against different classes of toxic compounds [14]. ABC proteins have also been implicated in mRNA translation and ribosome biogenesis. Thus their role goes beyond functions in membrane transport.

Only few ABC proteins from other fungal species have been functionally characterized, except for those mainly reported to be involved in multidrug resistance in human pathogens such as *Candida albicans *[19-23], *Aspergillus fumigatus *[24,25], and *Cryptococcus neoformans *[26-28], the model filamentous fungus *Emericella nidulans *(anamorph *Aspergillus nidulans*) [29-32], and the plant pathogen *Magnaporthe grisea *[33-35] (also see [36] for a review). On the other hand, recent sequencing projects unearthed an astonishing diversity of ABC proteins in fungal genomes, providing a unique opportunity for the comparative analysis of ABC proteins within this group of organisms. Previous attempts of phylogenetic analysis of fungal ABC proteins were either focused on a rather narrow group of species [37,38] or on a particular subfamily of ABC proteins [39,40]. Up to this date, a complete analysis has not been performed.

**Figure 2:**
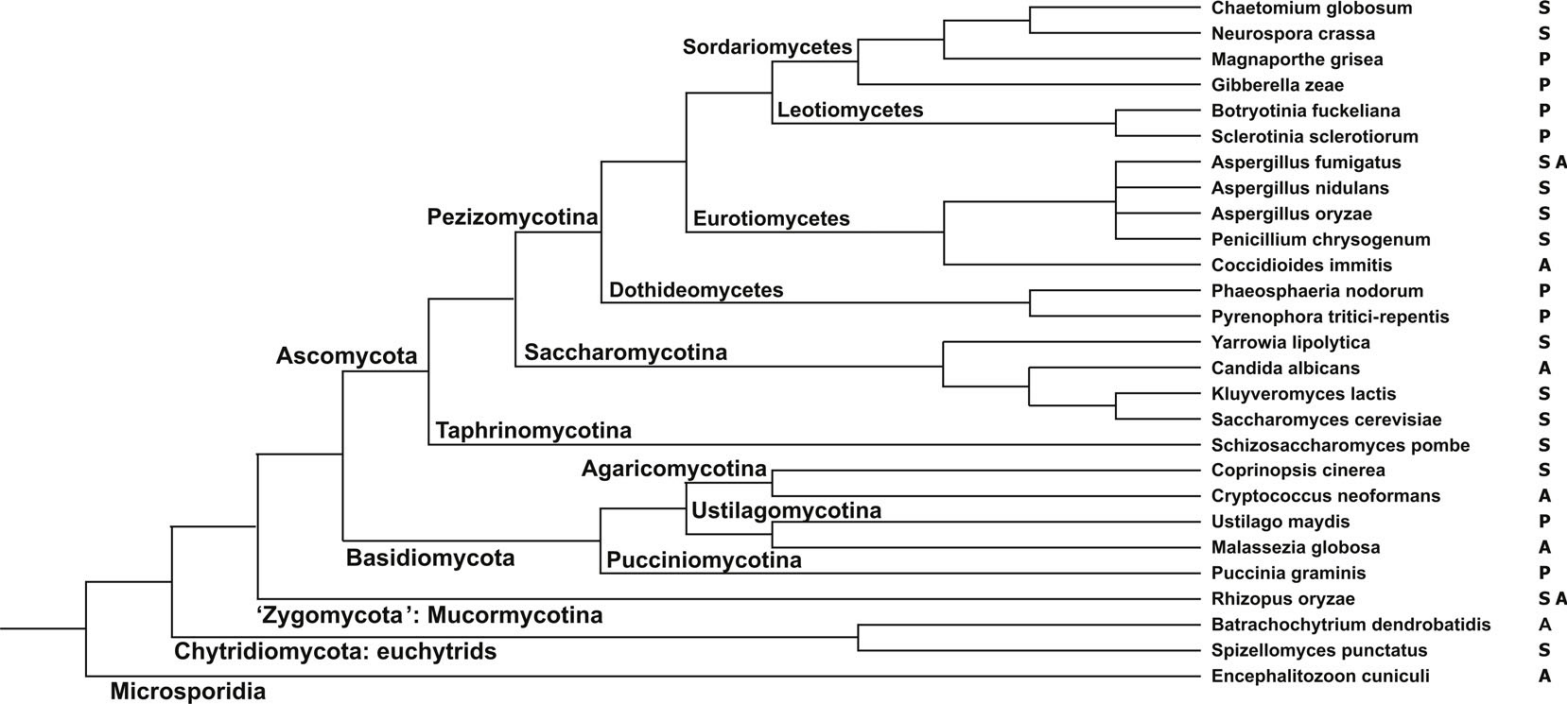
**The scheme illustrating phylogenetic positions of the analyzed fungal species**. The scheme is based on the analysis performed by [41]. Letters next to the species names indicate their life style. Saprophytic species are shown with S, animal pathogens with A, and plant pathogens with P.

Trying to fill this gap, we analyzed a representative set of fungal genomes for their content and distribution of ABC proteins. For this purpose, species were included such that all major groups of the fungal kingdom are covered, with the requirement that the completed genome sequences are publicly available (Figure 2) [41]. In total, we analyzed the genome sequences of 27 species representing five phyla and eighteen orders of fungi.

## Results and Discussion

### Identification of fungal ABC proteins

A set of 27 fungal species representing major evolutionary lineages within fungal kingdom [41] was used in our analysis. They were selected in order to create a representative set that covers the diversity of modern fungi as much as possible. So, within the defined orders of ascomycetes, the preference was given to the phylogenetically most distantly related species and, when available, to the species with different life styles (i.e, soil fungi, plant or animal pathogens). We also included in our analysis important model organisms (*S. cerevisiae*, *S. pombe*, and *A. nidulans*) and species from which at least some ABC proteins were characterized before.

The ABC proteins from the selected genomes were identified by multiple BLAST searches against the selected genome sequences. Members of every known subfamily of ABC proteins were used as queries for these searches. All BLAST hits with the E-value below 10^-4 ^were taken into subsequent analysis. This cut-off value was determined empirically as it allowed us to separate ABC proteins *sensu stricto *from more distantly related ATPases such as Rad50, SMC (Structural Maintenance of Chromosomes) and MSH (MutS Homologues) proteins. ABC proteins have in common that they all contain a nucleotide-binding domain with the characteristic Walker A and Walker B motifs. Thus this also led to the identification of Rad50, SMC and MSH proteins in our searches. However, members of the last three groups are treated separately as they do not represent transporters and for this reason they were not included in our analysis.

Similar results were obtained when instead of the full-length proteins, the ABC domains were used as BLAST queries. The hits with the highest score included proteins belonging to the same subfamily as a one used as a query. Numerous members of other subfamilies also had E-values well above the threshold. Other proteins containing nucleotide-binding domains were often identified in such searches as hits with a score below the threshold. Different results were obtained when only the transmembrane domains of ABC transporters were used as BLAST queries. These searches generally identified only proteins belonging to the same subfamily indicating that the degree of conservation of the transmembrane domains between different subfamilies of fungal ABC proteins is low.

The use of the members of all known ABC subfamilies allowed us to unequivocally identify all proteins of interest as well as fragments of ABC proteins less than 200 amino acids in size (Additional file 1). Taking this into account, we conclude that our searches resulted in the identification of a complete set of ABC proteins from the analyzed fungal genomes. As will be discussed below, in several cases certain ABC transporters were absent from the analyzed genome(s), whereas in other cases, the transporter appeared to be 'missing' because the corresponding genes contained gaps as these concerned nearly completed, but as yet unfinished genome sequences.

In total, 1109 predicted ORFs encoding putative ABC proteins were identified (Additional files 1, 2 and 3). The HUGO-approved scheme was used to classify the identified ABC transporters, and members of all subfamilies of eukaryotic ABC transporters were identified except for the ABC-H subfamily. The number of ABC proteins per genome varied by more than 5-fold between different species (Additional files 2 and 3) and is the largest in members of the subphylum *Pezizomycotina*. In the next sections, the results of our analysis are discussed for each subfamily.

### Subfamily A

ABC-A subfamily members were previously reported to be present in the genomes of animals, plants, and various protists (*Dictyostelium*, oomycetes, trypanosomes and entamoebae), but are absent in yeasts [7,12,42,43]. A characteristic feature of these proteins is the presence of a regulatory domain with multiple phosphorylation sites following the first NBF and a large extracellular loop between the first two transmembrane helices in the TMD (Figure 1) [44]. Both full-length and half-size transporters of the ABC-A class exist, although the half-size transporters appear to be absent from animal genomes. ABC-A transporters of vertebrates are the best characterized members of this group. They play a crucial role in lipid transport and metabolism, and have for instance been implicated in high-density lipoproteins biogenesis, lung surfactant production, retinal integrity and skin keratinization [45]. Currently, for none of the fungal ABC-A proteins their exact transport function is known.

**Figure 3:**
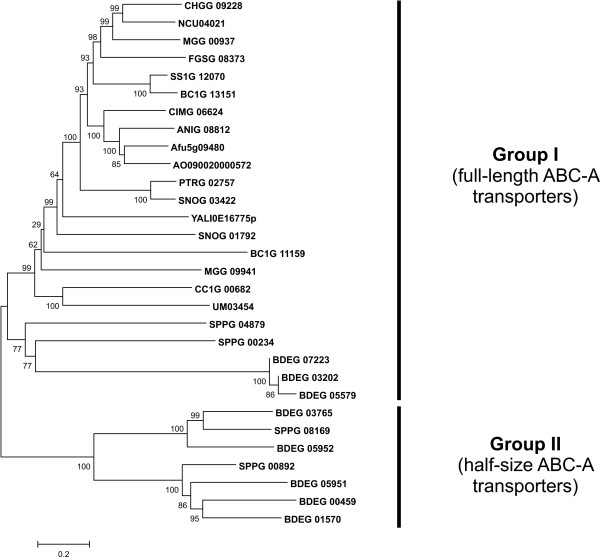
**Phylogenetic tree of fungal ABC-A proteins**. Predicted amino acid sequences of analyzed proteins were aligned, and the alignment was used to generate a phylogenetic tree using the neighbour-joining method. Numbers next to the branching points indicate the relative support from 500 replicates. Analysis was performed with MEGA 4.0.2 software package.

Our analysis revealed an uneven distribution of ABC-A proteins among fungi (Additional file 2) indicating multiple loss events during fungal evolution. We could not find members of this group in the genomes of *S. cerevisiae *and other species of the order *Saccharomycetales *(with a remarkable exception of *Y. lipolytica*), and also none in the genomes of *E. cuniculi*, *R. oryzae*, *S. pombe*, *P. chrysogenum *and the basidiomycetes *C. neoformans*, *M. globosa *and *P. graminis*. Most of the ABC-A proteins identified in the genomes of the remaining species are full-length transporters (Group I) (Figure 3). Half-size ABC-A proteins (Group II) were exclusively limited to the two species of chytrid fungi included in our analysis, *S. punctatus *and *B. dendrobatidis*, where they occur together with full-size transporters (Figure 3). It is generally accepted that chytridiomycetes belong to the most ancient fungi that are rooted close to the base of the fungal evolutionary tree. Thus, our observations indicate that both full-length and half-size ABC-A proteins must have been present in the genome of the common ancestor of modern fungi. The half-size transporters were likely lost in the lineage that led to the ascomycetes and basidiomycetes.

All four genes encoding ABC-A proteins in the genome of *S. punctatus *(two full-length and two half-size transporters) are apparently intact. At the same time, only five out of thirteen ABC-A genes in the *B. dendrobatidis *genome (BDEG_00459, BDEG_01570, BDEG_03765, BDEG_05951, and BDEG_05952) seem to encode for functional proteins (all half-size transporters). The other ABC-A proteins lack a transmembrane domain, which makes it unlikely that they are involved in membrane transport.

The genomes of the other species included in our analysis do not contain half-size ABC-A transporters, and mostly contain just a single gene for a full-length ABC-A transporter. The ascomycetes *M. grisea*, *B. fuckeliana *and *P. nodorum *contain two full-length ABC-A encoding genes (Additional file 2).

The absence of ABC-A transporters in a significant proportion of the analyzed genomes suggests that fungal ABC-A proteins are not essential for viability. They might be required under specific physiological conditions. Indeed, the only fungal ABC-A transporter which was functionally characterized so far, the *M. grisea *Abc4 protein (MGG_00937), is required for pathogenesis and appressoria formation [34]. A function in lipid transport and metabolism seem probable for fungal ABC-A proteins based on functional studies performed with mammalian homologs, but further studies are required to fully understand their role and reveal the identity of their substrates. The observed distribution also indicates that a loss of ABC-A genes occurred several times independently in the different fungal lineages. Both the highest number and diversity of ABC-A proteins is observed in the ancient chytrid species, while many of the more advanced fungi are completely devoid of ABC-A genes. Remarkably, these genes are absent from the compact genomes of yeast-like species, but also from much larger genomes of for instance *R. oryzae *and *P. graminis*. Therefore, genome size reduction was not the only driving force causing the elimination of ABC-A genes.

### Subfamily B

ABC-B proteins represent a large group of ABC proteins that includes both full and half-size transporters. These systems are widely distributed among eukaryotes, and are functionally diverse. They are for instance involved in multidrug resistance, antigen processing, mitochondrial peptides and pheromone export, Fe/S cluster proteins biogenesis and heavy metal resistance. Undoubtedly, the most well-known members of this group are mammalian multidrug transporters, such as P-glycoprotein (Pgp) that is involved in drug resistance in cancer cells [46]. Recently, the elucidation of the crystal structure of the mouse P-glycoprotein [47] provided important clues on the structural basis of the remarkable broad substrate specificity of these transporters. Eventually, structural and functional studies will result in the design of novel Pgp antagonists. Plant members of this group are involved in the export of auxins, secondary metabolites, and xenobiotics [12,48]. Interestingly, at least two plant ABC-B proteins appear to function as importers or uptake systems, a property that is very unusual for eukaryotic ABC transporters [49-51].

Both the full and half-size ABC-B transporters are abundantly distributed among the fungal genomes. While there is relatively little variation in the number of half-transporters between members of different fungal groups, full-size ABC-B proteins have undergone an extensive amplification, which is especially remarkable in the members of the subphylum *Pezizomycotina*.

Fungal full-size ABC-B transporters have been characterized to quite some detail during the last decades. Clearly, the *S. cerevisiae *protein Ste6p (YKL209c) is the best characterized member of this group, being the first ABC transporter identified in yeast [52,53]. Ste6p is haploid-specific, and required for the export of the farnesylated peptide pheromone known as **a**-factor. ScSte6p orthologues in *S. pombe *(Mam1, SPBC25B2.02c) and *C. albicans *(Hst6, CaO19.7440) fulfill a similar function [54,55].

Not surprisingly, a number of fungal full-length ABC-B proteins are involved in multidrug resistance, as, for example, *S. pombe *Pmd1 (SPCC663.03), *A. fumigatus *Mdr1 (Afu5g06070), or *A. nidulans *AtrD (ANIG_02300) [25,30,56]. Additionally, a role in β-lactam secretion was proposed for the *A. nidulans *AtrD transporter [30]. The physiological significance of ABC-B transporters is not limited to drug extrusion and pheromone export only. The *M. grisea *Abc3 transporter (MGG_13762) is involved in the oxidative stress response, and is required for host penetration [35], while the *A. fumigatus *AbcB (Afu3g03430) protein was demonstrated to be involved in the excretion of siderophore peptide breakdown products [57]. The latter is of special interest as a number of fungal ABC transporters are closely associated with nonribosomal peptide synthetase (NRPS) clusters [58] and, therefore, might have a role in the secretion of peptide-like secondary metabolites.

**Figure 4:**
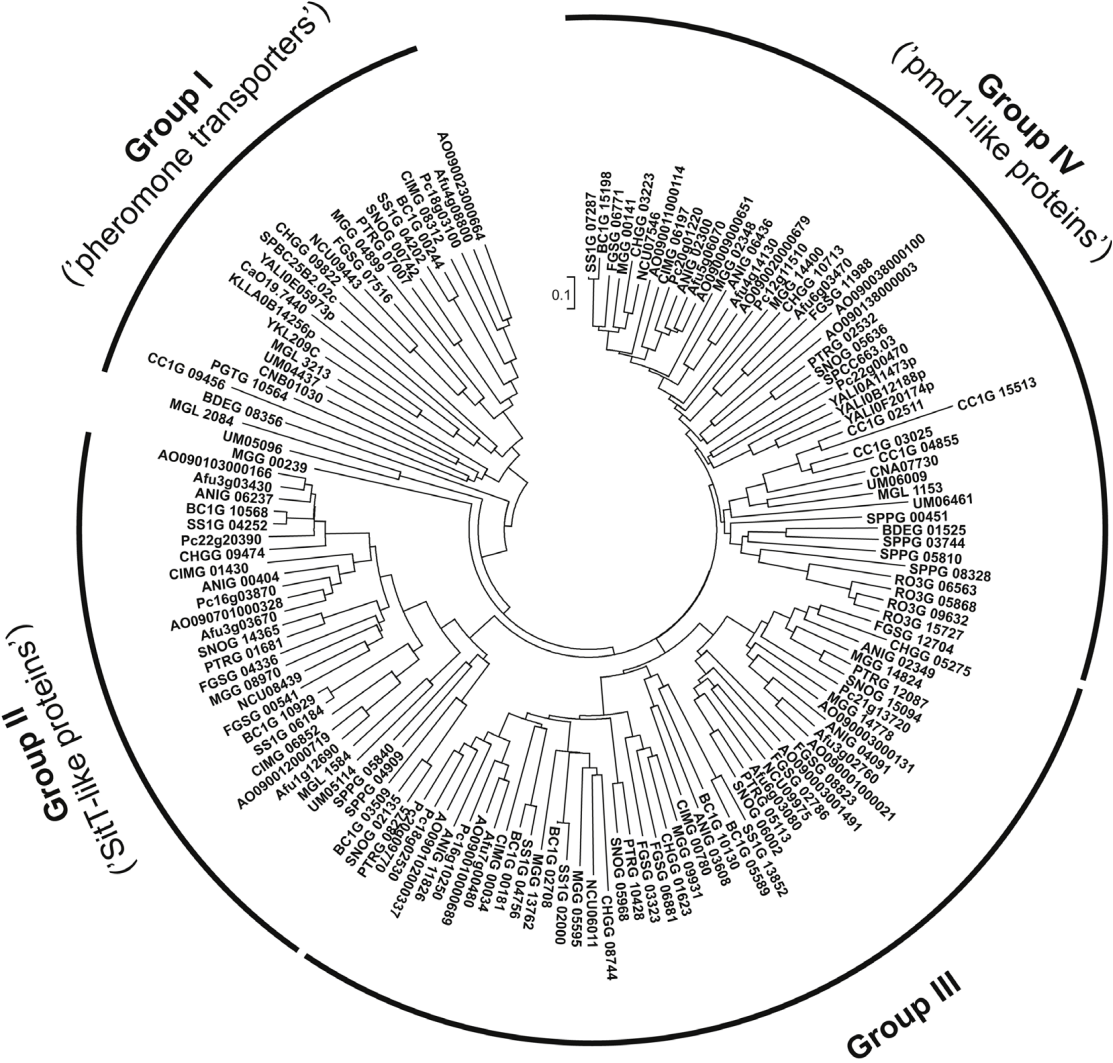
**Phylogenetic tree of fungal full-length ABC-B proteins**. The tree was generated as described in the legend to the Figure 3.

Full-size ABC-B transporters are present in all analyzed fungal species except for *E. cuniculi*. Phylogenetic analysis separates them in several clusters that are briefly discussed below (Figure 4, Additional file 4). The first group (Group I, "pheromone transporters") consists of ScSte6 orthologues, presumably involved in sex pheromone export. Its members were found in all analyzed species except for *E. cuniculi*, *S. punctatus*, *R. oryzae*, and *A. nidulans*. The absence of the pheromone transporter from the *A. nidulans *genome is quite surprising, as the corresponding gene was identified in all other analyzed ascomycetous genomes, including *A. fumigatus *and *A. oryzae*. Its absence might be explained by the homothallic nature of *A. nidulans*. The remainder of the analyzed genomes contain only one gene belong to this group, with the exception of *C. cinerea *that has two identical copies of an ABC-B transporter on supercontig #6. Pheromone transporters are the only full-size ABC-B transporters identified in the genomes of *S. cerevisiae*, *K. lactis*, *C. albicans*, and *P. graminis*. The *B. dendrobatidis *protein BDEG_08356 was placed next to this group in some of the constructed trees, but this suggestion is not supported by bootstrap analysis. Therefore, it remains uncertain whether this protein should be include into this group.

Members of the second cluster (Group II) were found mainly in the subphylum *Pezizomycotina *but also in chytrid *S. punctatus *and basidiomycetes like *U. maydis *and *M. globosa*. Their number ranges from 1 to 3 genes per genome. The *A. fumigatus *SitT transporter (Afu3g03430) (also known as AbcB) is required for the excretion of fusarinine breakdown products [57]. Interestingly, the fusarinine biosynthesis clusters in the genomes of other *Pezizomycotina *also contains transporters closely related to *A. fumigatus *SitT, suggesting a conserved function [57,58].

The next group of full-size ABC-B transporters (Group III) is limited in its distribution to species of the subphylum *Pezizomycotina*. They contain up to five members of this subgroup. Little is known about their functional role. The expression of the *A. nidulans *AtrC (ANIG_03608) transporter increases in the presence of cycloheximide, but the corresponding gene deletion mutant had no obvious phenotype or sensitivity towards cycloheximide [30,59]. Another member of this group, *M. grisea *Abc3 (MGG_13762), is required for host cell penetration, but the underlying mechanisms remain unknown [35].

The last cluster recognized in our analysis (Group IV) consists of proteins showing similarity with the *S. pombe *Pmd1 (SPCC663.03) transporter. Members of this cluster are found in the genome of most of the analyzed species with the exception of *E. cuniculi*, *S. cerevisiae*, *K. lactis*, *C. albicans*, and *P. graminis*. The number of genes belonging to this group ranges between one and five per genome. All fungal ABC-B proteins with a known function in multidrug resistance belong to this cluster, but the exact function of the majority of these proteins is unknown.

In contrast to full-size ABC-B proteins, the functions of the ABC-B half-transporters are not linked to the multidrug resistance. Two major subgroups are usually recognized: HMT (heavy metal tolerance) and MPE (mitochondrial peptide exporter). All fungal ABC-B half-transporters characterized so far are proteins that localize either in the mitochondrial or vacuolar membrane.

**Figure 5:**
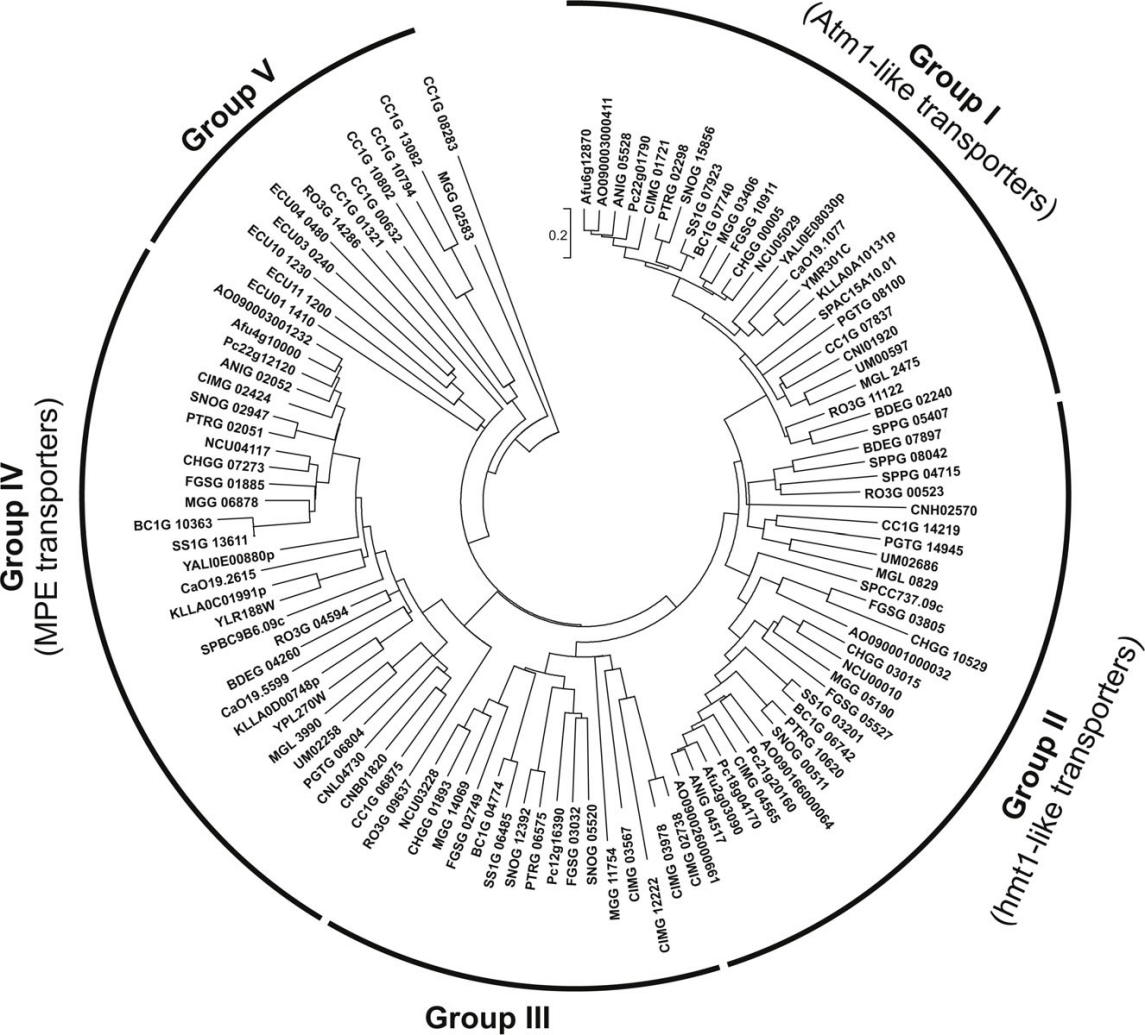
**Phylogenetic tree of fungal half-size ABC-B proteins**. The tree was generated as described in the legend to the Figure 3.

HMT transporters were found in the genomes of every analyzed species except for *E. cuniculi *(Figure 5, Additional file 5). Iconic members of this group are the *S. cerevisiae *Atm1p (YMR301C) and *S. pombe *Hmt1 (SPCC737.09c) proteins. *S. cerevisiae *Atm1p localizes to the inner membrane of mitochondria, where it exports precursors of iron-sulfur (Fe/S) clusters synthesized in the mitochondria and exported into the cytosol [60-62]. The function of Atm1p in the biogenesis of Fe/S clusters is highly conserved in the evolution, as homologous proteins of plants and animals have a similar role [63,64]. A single copy of the corresponding gene was found in the genome of every species included in our analysis except for *E. cuniculi *(Group I on Figure 5).

Another well-studied transporter of this group, the *S. pombe *Hmt1, resides in the vacuolar membrane and is required for the cadmium tolerance. It is able to transport both phytochelatin- and glutathione-cadmium complexes, contributing in this way to the sequestration of these molecules in the vacuole [65-67]. The function of Hmt1 is evolutionary conserved, as homologous proteins were demonstrated to be involved in cadmium tolerance in *Caenorhabditis elegans *[68], *Chlamydomonas reinhardtii *[69] and *Drosophila melanogaster *[67]. Homologues of the *S. pombe *Hmt1 (Group II) were found in the genomes of most of the analyzed species, with the exception of *E. cuniculi *and members of the order *Saccharomycetales*. The copy number of Hmt1-related genes in different fungi varies between one and three per genome, so it cannot be excluded that the paralogous proteins fulfill alternative functions.

Finally, a small group of Hmt-related transporters (Group III on the Figure 5) is specific for the subphylum *Pezizomycotina*. One or two corresponding genes were identified in most of the analyzed species. As many as six genes were found in *C. immitis*, four of which, however, represent putative pseudogenes. Proteins belonging to this group are apparently absent from the genomes of *P. chrysogenum *and *Aspergillus *species. The biological function of the members of this cluster currently remains unknown.

MPE transporters constitute a second group of half-size ABC-B transporters in fungal genomes. Their prototype is the *S. cerevisiae *protein Mdl1p (YLR188W). This protein localizes to the inner mitochondrial membrane and is required for the export of peptides released upon proteolysis of inner-membrane proteins by the m-AAA protease [70]. It also plays a role in the regulation of cellular resistance to oxidative stress [71]. The function of the related *S. cerevisiae *protein Mdl2p (YPL270W) is less well understood. MPE transporters are ubiquitously present in all species included in our analysis except for *E. cuniculi *and *S. punctatus*. There is only one MPE transporter in most of the analyzed fungi. The presence of two MPE genes in the genomes of *R. oryzae*, *C. neoformans*, and hemiascomycetous yeasts *S. cerevisiae*, *K. lactis *and *C. albicans *most likely resulted from independent lineage-specific duplication events.

Unclassified ABC-B half-transporters not placed within any of the above-described groups were assigned to Group V (Figure 5). This group includes, among others, six *E. cuniculi *transporters, two of which (ECU01_0200 and ECU01_1410) are identical. Notably, five of those *E. cuniculi *proteins contain putative mitochondrial pre-sequences [72] suggesting a localization to the mitosomes, mitochondrial remnants of microsporidia. The function of the *E. cuniculi *ABC-B transporters is currently unknown.

Group V also includes six *C. cinerea *proteins showing some similarity to the bacterial HlyB/MsbA transporters. Related proteins were also found in the genome of some other basidiomycetous fungi (data not shown). The function of these proteins has not been addressed so far. There is also one *R. oryzae *and one *M. grisea *protein placed within group V.

### Subfamily C

ABC-C proteins are full-length transporters found in all major groups of eukaryotes. Many, but not all contain an additional N-terminal hydrophobic region (Figure 1). Some ABC-C transporters are involved in the detoxification of toxic compounds by means of their extrusion from the cell or sequestration in the vacuole. However, the substrates include conjugates of drugs with organic anionic molecules like glutathione and glucuronide rather than the drugs themselves. Fungal ABC-C proteins, except for the *S. cerevisiae *transporters, are poorly characterized. Notably, some of the animal ABC-C transporters are not primarily active transporters, as they function either as ATP-gated chloride channels (the cystic fibrosis transmembrane conductance regulator CFTR (ABCC7)) or as potassium channel regulators (sulfonylurea receptors SUR1 (ABCC8) and SUR2 (ABCC9)) [7]. No such functions were reported for fungal ABC-C proteins so far.

**Figure 6:**
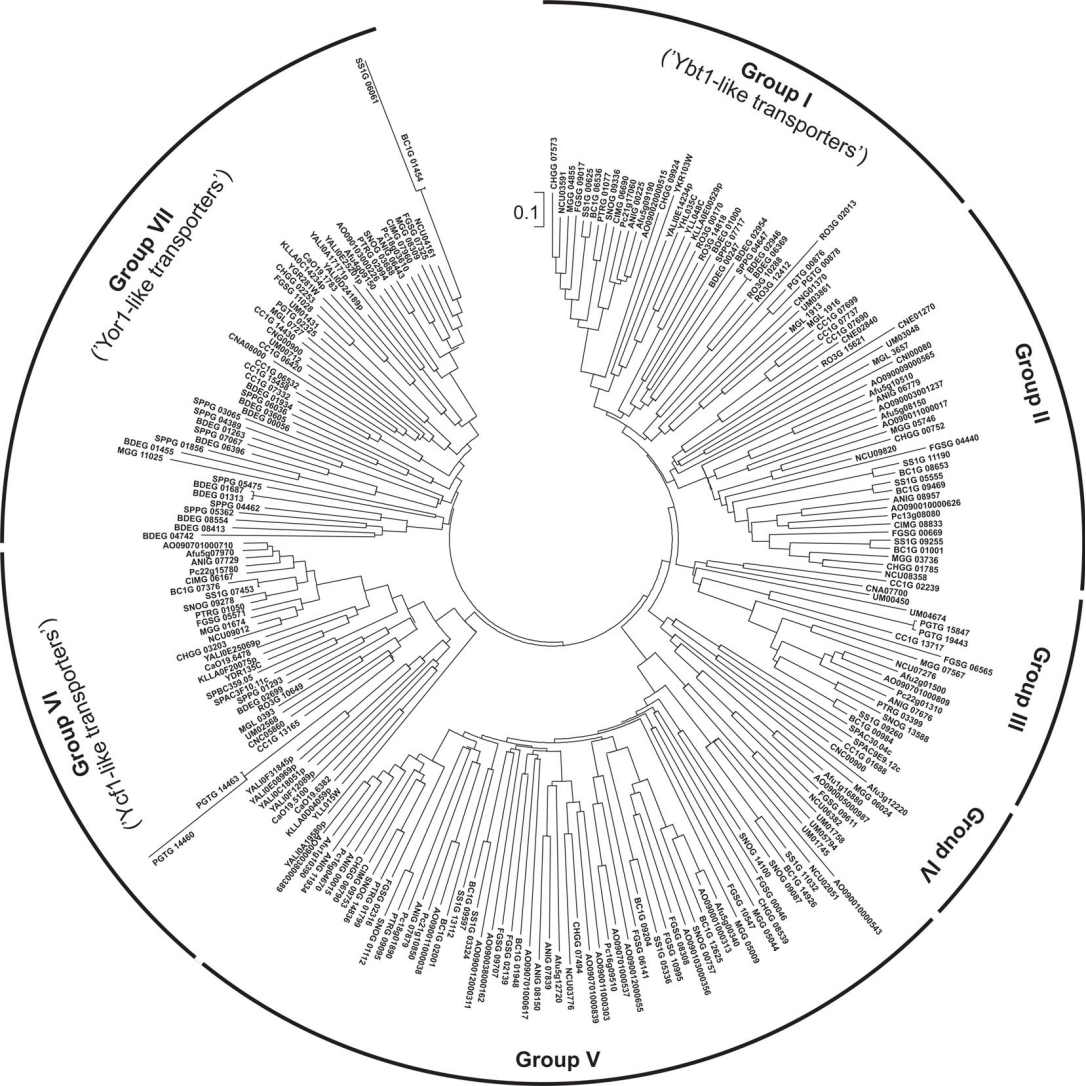
**Phylogenetic tree of fungal ABC-C proteins**. The tree was generated as described in the legend to the Figure 3. *A. nidulans *transporters clustered with secondary metabolism genes are marked with asterisks.

Members of the ABC-C subfamily are found in the genomes of all analyzed species except for *E. cuniculi*. As many as seven phylogenetic clusters exist (Figure 6, Additional file 6). Three include *S. cerevisiae *proteins, and an additional cluster contains the *S. pombe *transporters. The three remaining groups contain no proteins with known functions. One of these (group III in Figure 6) is specific for *Pezizomycotina *and basidiomycetes, and there is a single corresponding gene in the genomes of ascomycetes and *C. neoformans*, and two genes in other basidiomycetes. Three of the analyzed species (*C. immitis*, *Ch. globosum *and *M. globosa*) lack members of this group.

Group II includes transporters of *R. oryzae*, *Pezizomycotina *species (except for *P. tritici-graminis *and *P. nodorum*) and basidiomycetes (with the exception of *P. graminis*). Their number ranges from one to five per genome, with the highest number in *A. oryzae*. Group V is present in the genomes of *Pezizomycotina *and *U. maydis*, and their number per species ranges from two to up to thirteen. Again, the highest number was observed in *A. oryzae*, but some are putative pseudogenes. Remarkably, at least four out of the seven *A. nidulans *proteins belonging to this group are associated with secondary metabolism clusters. ANIG_00015, ANIG_11934 (previously known as AN1240), and ANIG_07879 are found in the immediate vicinity of NRPS genes, and ANIG_07839 is located next to the putative polyketide synthase (PKS)-like enzyme [58]. The structure of some of those clusters is highly conserved among aspergilli, and it seems very likely that those transporters are involved in the export of the corresponding secondary metabolites.

The presence of yeast transporters within the remaining clusters of the ABC-C subfamily provides at least some clues about the possible functions of these proteins. Group I contains transporters with similarity to *S. cerevisiae *Ybt1p (YLL048C). This vacuolar protein transports bile acids [73], but its physiological role is not fully understood. Two of the other *S. cerevisiae *proteins belonging to this cluster, i.e., Vmr1p (YHL035C) and Nft1p (YKR103W) are largely uncharacterized. There is no information about the function of Ybt1p-related transporters from other fungi available. Ybt1p homologues appear absent from the genomes of *S. pombe*, *C. albicans *and *C. cinerea*. There is a single Ybt1p orthologue in most of the remaining ascomycetes, with the exceptions of *S. cerevisiae *(three genes) and *Ch. globosum *(two genes), whereas members of the other fungal phyla contain up to five Ybt1-like genes, such as in *R. oryzae *and *B. dendrobatidis*.

Group IV is a small collection of fungal ABC-C proteins that in our analysis only contains a total of ten members. There is a single representative in the genomes of *A. oryzae*, *G. zeae*, *M. grisea*, *N. crassa*, *C. cinerea *and *C. neoformans*, whereas both *S. pombe *and *A. fumigatus *have two of these systems. A vacuolar localization was demonstrated for the *S. pombe *Abc4 (SPAC30.04c). The *S. pombe *Abc1 (SPAC9E9.12c) protein localizes to ER-like membranes, but this might be due to a mislocalization as a result of the overexpression [15]. The *S. pombe *Abc4 protein together with the Abc2 transporter were shown to be involved in the export of the red-colored purine intermediate in the *ade1 *mutant strain [15], but its function under normal physiological conditions remains unclear.

Group VI includes ABC-C transporters related to *S. cerevisiae *Ycf1p (YDR135C) and Bpt1p (YLL015W) proteins. Those two yeast transporters localize to the vacuolar membrane and are involved in the detoxification of heavy metals via transport of glutathione conjugates. They are also able to transport unconjugated bilirubin [74-76]. Ycf1p homologues were identified in every species included in our analysis except for *E. cuniculi*. While most of the fungi have only a single Ycf1 homologue, there are two Ycf1-related transporters in the *S. pombe *and *P. graminis *genomes. An especially remarkable expansion of this family occurred in the *Saccharomycetales *lineage. The genomes of *S. cerevisiae *and *K. lactis *contain two members of this group, *C. albicans *contains three members, and as many as six are present in *Y. lipolytica*. One of the *C. albicans *transporters belonging to this group, Mlt1 (CaO19.5100), was demonstrated to be involved in virulence [77].

Members of group VII are closely related to the *S. cerevisiae *Yor1p (YGR281W). This plasma membrane-localized transporter mediates export of many different organic anions including oligomycin as well as phospholipids [78-80]. Disruption of the *yor1 *gene causes an increased sensitivity to a variety of drugs and xenobiotics. Yor1 homologues were not found in the genomes of *R. oryzae *and *S. pombe*. Most of other ascomycetes and the basidiomycetes *M. globosa *and *P. graminis *contain a single Yor1-like transporter, but *G. zeae *and *M. grisea *have two genes. *Y. lipolytica *even contains three Yor1-like transporters. The genomes of the basidiomycetes *U. maydis *and *C. neoformans *contain two Yor1 homologues, while five members of this group were identified in *C. cinerea*. Finally, two chytrid species, *B. dendrobatidis *and *S. punctatus*, contain as many as eleven and eight Yor1-like transporters, respectively. None of the proteins belonging to this cluster has been characterized so far except for the *S. cerevisiae *Yor1p itself.

### Subfamily D

ABC-D transporters of animals, fungi and protists are half-size transporters that localize to the peroxisomal membrane. They mediate the import of long-chain fatty acids. Both full and half-size ABC-D proteins were identified in plant genomes. The *A. thaliana *Abcd2 (Pmp1) protein contains a putative chloroplast transit peptide, and has been suggested to be plastid localized [12,81]. *S. cerevisiae *has two proteins that belong to this group, Pxa1p (YPL147W) and Pxa2p (YKL188C). These proteins function as a heterodimer. Corresponding mutants are unable to grow on fatty acids such as palmitate or oleate as a sole carbon source [82-84].

**Figure 7:**
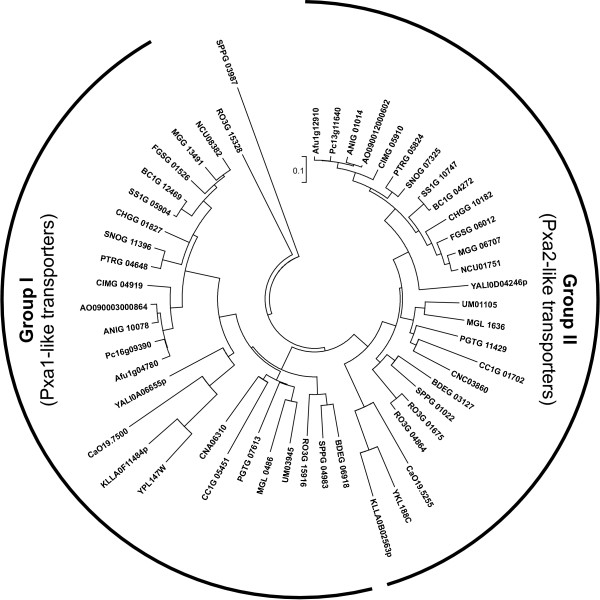
**Phylogenetic tree of fungal ABC-D proteins**. The tree was generated as described in the legend to the Figure 3.

ABC-D transporters are found in every species included in our analysis except for *E. cuniculi *and *S. pombe*. While *E. cuniculi *lacks peroxisomes [85], a set of PEX genes required for peroxisome biogenesis was identified in the genome of *S. pombe *[86], and peroxisomes were also observed in budding yeast cells [87]. Therefore, the lack of ABC-D transporters in *S. pombe *is quite unexpected. The presence of this set of ABC-D transporters in other fungi is a conserved feature (Figure 7). The only two exceptions are *S. punctatus *with three ABC-D transporters and *R. oryzae *that contains four homologs and one putative pseudogene.

Phylogenetic analysis separates fungal ABC-D transporters into two groups with the *S. cerevisiae *transporters Pxa1p and Pxa2p as their prototypic members (Figure 7, Additional file 7). Only two transporters in our analysis, *S. punctatus *SPPG_03987 and *R. oryzae *RO3G_15328, were placed outside of these well-defined groups. It would be of interest to investigate in details whether the function of these two particular transporters is different from those described for other ABC-D proteins.

### Subfamilies E and F: soluble ABC proteins

Both the ABC-E and ABC-F proteins lack transmembrane domains and consist solely of two nucleotide-binding domains. Their function within the cell is not related to transport. Nevertheless, the crucial role of the members of these groups is emphasized by the fact that three of them (Rli1, Arb1, and Yef3) are essential for viability of *S. cerevisiae *cells [16].

**Figure 8:**
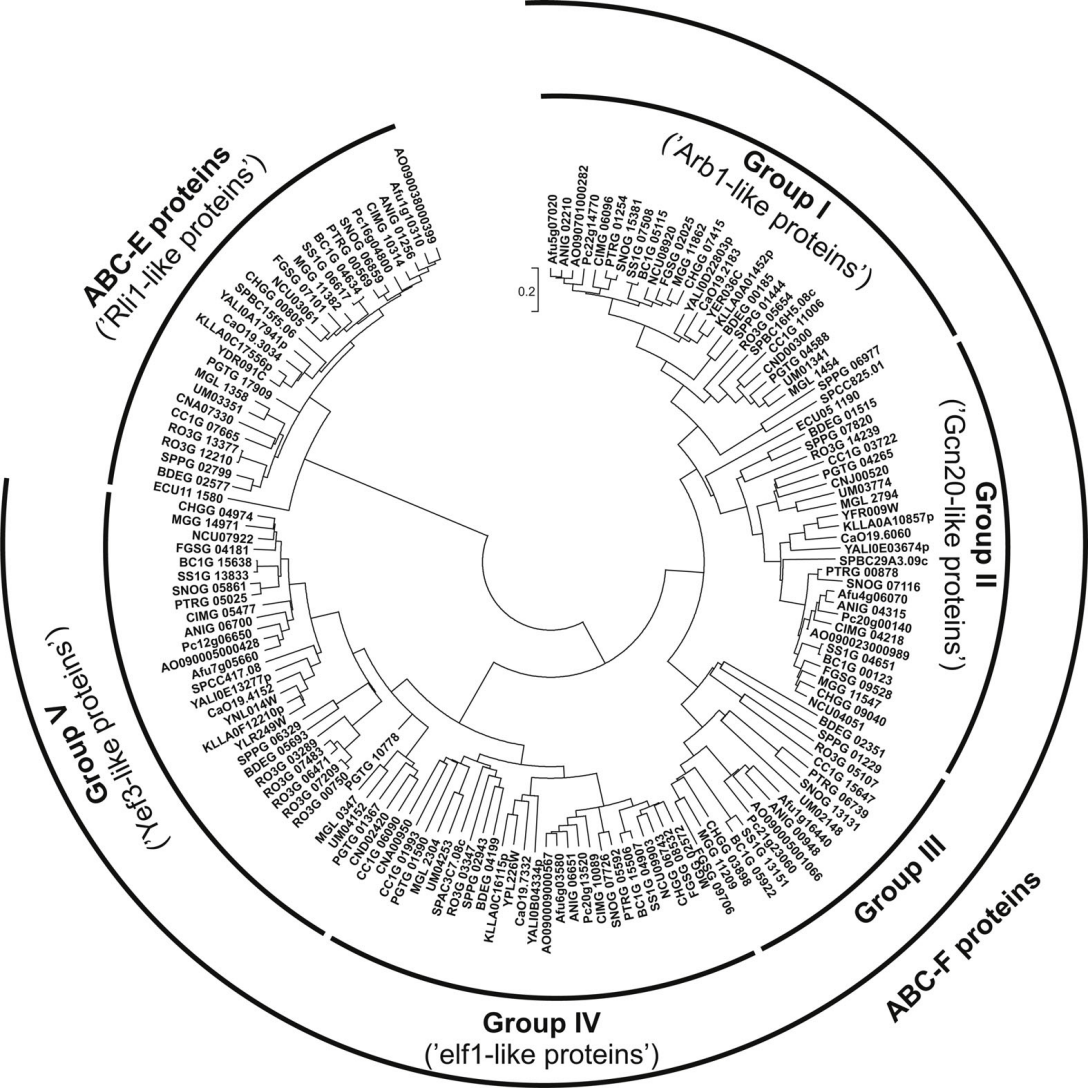
**Phylogenetic tree of fungal ABC-E and ABC-F proteins**. The tree was generated as described in the legend to the Figure 3.

ABC-E proteins were identified in the genomes of both eukaryotes and archaea. Most of the eukaryotic genomes analyzed so far have a single member of this group, although plants contain two. The prototypical ABC-E protein, *S. cerevisiae *Rli1p (YDR091C), is an essential iron-sulfur protein required for ribosome biogenesis and translation initiation [88,89]. A single corresponding gene was found in every species included in our analysis (Figure 8, Additional file 8), except for *R. oryzae*, were two highly similar genes (93% identity on nucleotide level) exist, that likely emerged from a recent duplication. The uniforms distribution of ABC-E proteins among fungi and other eukaryotes supports the notion that they are involved in a highly conserved activity in the cell.

The functions of ABC-F proteins are related to different aspects of translation, as they are involved in ribosome biogenesis, translational control, mRNA export, or act as translational elongation factors. Most of the analyzed fungal genomes have either four or five members of this group, however, there is only a single ABC-F protein in *E. cuniculi *whereas as many as ten genes were identified in *R. oryzae *genome.

Phylogenetic analysis separated the fungal ABC-F proteins into five groups (Figure 8, Additional file 8). The Group I of fungal ABC-F proteins is related to the *S. cerevisiae *Arb1p (YER036C) protein. This essential protein is involved in 40S and 60S ribosome biogenesis [90]. Most of the fungal genomes harbor a single Arb1p orthologue, but the gene is absent in the *E. cuniculi *genome while *S. punctatus*, *R. oryzae *and *S. pombe *contain two Arb1 homologues.

The *S. cerevisiae *Gcn20p protein (YFR009W) is a prototypical member of Group II and it functions as a positive regulator of Gcn2p kinase activity [91]. There is a single member of this group present in every analyzed genome. The only identified ABC-F protein of *E. cuniculi *also belongs to this group.

Members of Group III fungal ABC-F proteins are absent from the genomes of *S. cerevisiae *and *S. pombe*. In addition to these yeast species, none of the proteins belonging to this cluster were found in the genomes of *E. cuniculi*, other *Saccharomycetales *species, *C. immitis*, *N. crassa*, *C. neoformans*, *M. globosa *and *P. graminis*. There is a single member of the group present in the remaining species.

Representative members of Group IV are the *S. pombe *Elf1 (SPAC3C7.08c) and *S. cerevisiae *New1p (YPL226W). The fission yeast protein Elf1 is known to function as a mRNA export factor [92]. ScNew1p was not functionally characterized so far, but the presence of an Asn/Gln-rich region supports the formation of [NU+] prions. All analyzed species except for *E. cuniculi *contain a single Elf1-related protein.

Proteins of Group V are related to the *S. cerevisiae *Yef3p (YLR249W) and Hef3p (YNL014W) proteins. Both function as translation elongation factor 3 proteins that stimulate the binding of aminoacyl-tRNA to ribosomes [93,94]. Under normal laboratory conditions Hef3p is not expressed, and Yef3p is responsible for the EF3 function. Yef3p is essential for cell viability, while a *HEF3 *gene disruption has a little effect. In most of the analyzed species there is only a single Yef3-like protein, so *S. cerevisiae *with two EF3 proteins appears an exception among the fungi. The only other species with more than one member of this group is *R. oryzae *that contains four corresponding genes and one putative pseudogene. A putative pseudogene belonging to this group was also found in the genome of *A. fumigatus*.

### Subfamily G

The characteristic feature distinguishing ABC-G transporters from other subfamilies discussed above is their reverse topology, i.e. the nucleotide-binding domain precedes the transmembrane domain (Figure 1). Both full-length and half-size members of this subfamily are known, however, full-length ABC-G proteins are apparently absent from animal genomes. This group was intensively studied as several of these transporters are linked to pleiotropic drug resistance (PDR) phenomena. Hence, full-length ABC-G transporters are often referred to as PDR transporters. Extensive phylogenetic analysis of the fungal members of this group was published recently [39]. The half-size ABC-G proteins are sometimes called WBC transporters after the prototypical members of this group, *D. melanogaster *white-brown complex transporters required for eye pigment formation.

Ten ABC-G transporters are known from the genome of *S. cerevisiae*. Despite the fact that yeast PDR transporters were studied extensively during the last two decades, the functions of three are still unknown, i.e., Adp1p (YCR011C), Pdr18p (YNR070W) and YOL075C. Five *S. cerevisiae *transporters (Snq2p (YDR011W), Pdr5p (YOR153W), Pdr10p (YOR328W), Pdr11p (YIL013C), and Pdr15p (YDR406W)) are part of the PDR network and they contribute to the pleiotropic drug resistance by export of various, chemically unrelated hydrophobic molecules from the cell [95,96]. The Pdr12p (YPL058C) protein confers cell resistance to weak organic acids [97,98]. The function of PDR proteins is not limited to drug export, as Pdr11p and Aus1p (YOR011W), are required for sterol uptake and anaerobic growth [99]. Additionally, a role for two PDR proteins, Snq2p and Pdr5p, in yeast quorum sensing has been proposed recently [100]. ABC-G transporters described from other fungal species often contribute to drug resistance too (see [101] for a recent review). However, the physiological functions of PDR transporters might also relate to the translocation of various lipid molecules, as shown for *C. albicans *transporters [102].

**Figure 9:**
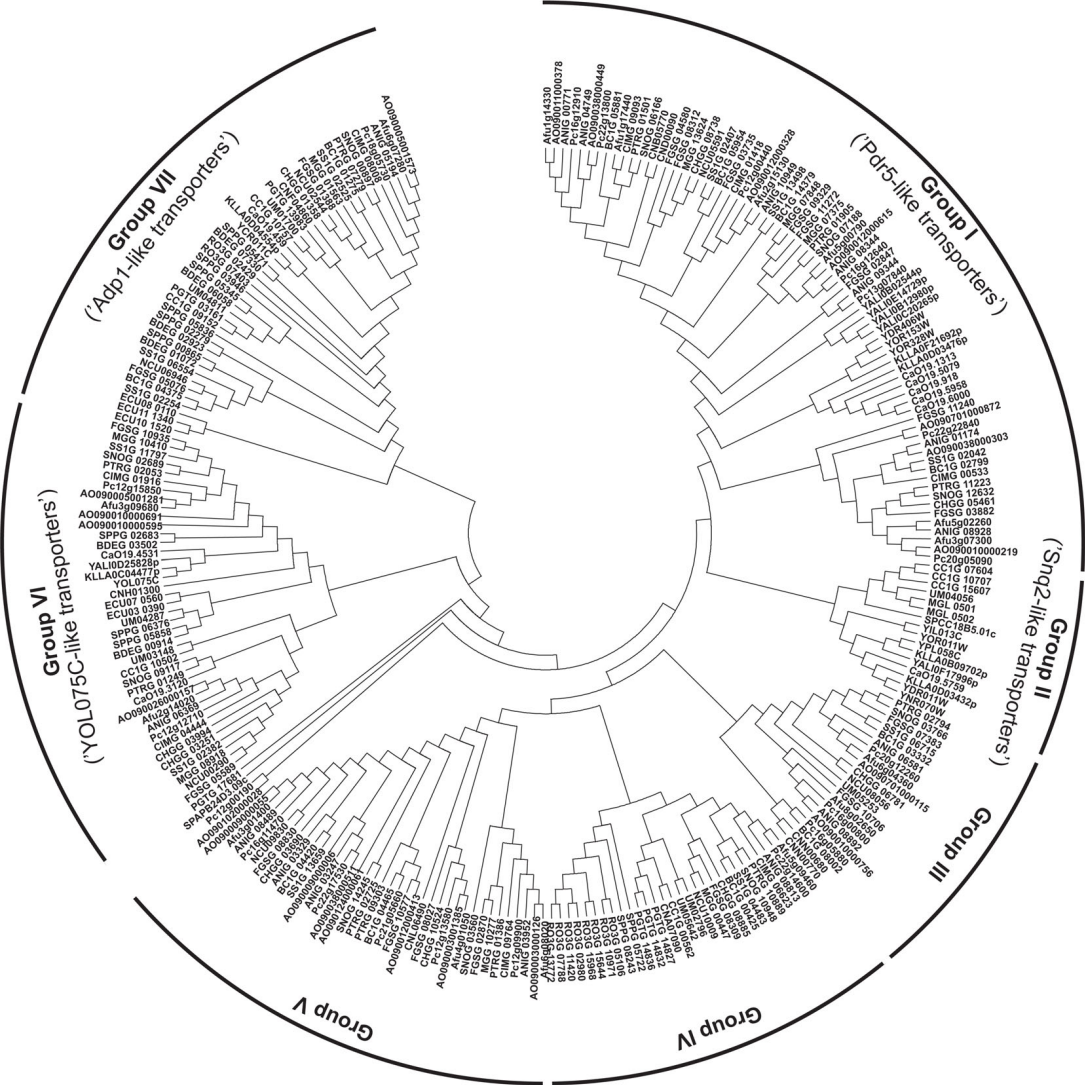
**Phylogenetic tree of fungal ABC-G proteins**. The tree was generated as described in the legend to the Figure 3.

A comprehensive phylogenetic analysis of fungal ABC-G transporters proved to be complicated by the fact that their massive expansion in fungal genomes apparently occurred after the diversification of the major fungal lineages. However, proteins related to the *S. cerevisiae *Adp1p and YOL075C form two groups that are clearly separated from the other fungal ABC-G transporters (Figure 9, Additional file 9).

Although Adp1 was one of the first yeast PDR transporters discovered [103], its function has remained enigmatic. This transporter is unusual as it has only one central NBD located between two transmembrane domains, and its first NBD is replaced by a large soluble domain containing EGF (Epidermal Growth Factor) repeats [16,17]. Adp1 also displays sequence similarity with the *D. melanogaster white*, *brown *and *scarlet *half-transporters involved in pigment precursor transport in the eye. Related proteins (Group VII in Figure 9) are found in most of the analyzed species with the exception of *S pombe*, *Y. lipolytica *and *M. globosa*. Their number ranges from a single copy in most ascomycetes to six genes in chytrid *S. punctatus*.

Similarly to Adp1p, the function of the *S. cerevisiae *YOL075C protein remains unknown. Related proteins (Group VI) were found in most of the species included in our analysis. However, they are apparently missing in the genomes of *R. oryzae*, *S. pombe*, *M. globosa *and *P. graminis*. Notably, some of fungal ABC-G transporters that belong to this group have the characteristic NBD-TMS_6 _topology of half-transporters (like CaO19.3120), while other like YOL075Cp are typical full-size transporters with the topology NBD-TMS_6_-NBD-TMS_6_. The number of members of this group in fungal genomes ranges from one to four, with the highest number observed in *A. oryzae*. Adp1- and YOL075C-related proteins are the only ABC-G transporters identified in the genomes of *E. cuniculi *and *B. dendrobatidis*. Taking into account an early origin and a wide distribution of these two groups in the fungal genomes, these transporters might perform some important but so far unknown function.

The 'true' PDR transporters were identified in most of the analyzed genomes, with the exception for the most basal species *E. cuniculi *and *B. dendrobatidis*. Their number per genome varies significantly ranging from two in *S. punctatus*, *S. pombe *and *M. globosa *to as many as seventeen in *A. oryzae *and *P. chrysogenum*. Previous observations [101,104] and our phylogenetic analysis indicates that PDR transporters are the least conserved among fungal ABC proteins, suggesting their rapid evolution after the divergence of the main fungal lineages. Because of multiple gene duplications and/or loss events, it is difficult to define the groups of orthologous PDR proteins in the different species. However, five clusters of PDR transporters could be recognized in our phylogenetic analysis (Figure 9, Additional file 9). All of them, except for Group IV, are restricted to the genomes of higher fungi (ascomycetes and basidiomycetes).

The largest Group I contains more than 70 members. Corresponding proteins are found in all ascomycetes except for *S. pombe*, but they are almost entirely missing from the analyzed basidiomycetes with *C. neoformans *as only exception. The number ranges from a single gene in *N. crassa *up to eight genes in *G. zeae*. These systems are also remarkably abundant in the genomes of *P. chrysogenum *and *Aspergillus *species. This group includes transporters with well-established roles in multidrug resistance as for instance the *S. cerevisiae *Pdr5p, Pdr10p and Pdr15p as well as the *C. albicans *Cdr1p, Cdr2p, Cdr3p, and Cdr4p proteins.

Group II is much smaller than Group I. Members are present only in *S. pombe*, species of the order *Saccharomycetales *and three of the analyzed basidiomycetes (*C. cinerea*, *U. maydis*, and *M. globosa*). This branch of the phylogenetic tree received relatively low support in the bootstrap analysis, so the members from ascomycetes and basidiomycetes might be treated as two separate groups. In different species, the number of the proteins belonging to this group ranges from one to five. The highest number is found in the genome of *S. cerevisiae*, including Snq2p, Pdr11p, Pdr12p, Pdr18p and Aus1p. The *S. pombe *brefeldin A efflux protein Bfr1 is also placed in this group.

Groups III and V are similar in their distribution as they are only found in the species of *Pezizomycotina *and basidiomycetes. Group III members are absent in *C. immitis *and *M. grisea*, but present in the two species of basidiomycetes, *U. maydis *and *C. neoformans*. Proteins belonging to Group V, on the other hand, were found in all analyzed species of *Pezizomycotina*, while the only representative among basidiomycetes is in *C. neoformans*. Members of Group III are less abundant and usually present as one or two copies (only *P. chrysogenum *has three of them). Up to seven transporters belonging to Group V could be found in *A. oryzae*.

Unlike the two previous groups, members of Group IV are also found outside of the higher fungi, namely in *S. punctatus *and *R. oryzae*. They are missing, however, from the genomes of *Saccharomycetales*, *M. globosa*, and, quite surprisingly, also absent in *A. oryzae*. In other ascomycetes they are universally present as a single copy, while there are two of such genes in *U. maydis *and *S. punctatus*, three were found in *P. graminis*, and as many as eight are present in *R. oryzae*.

Finally, four ABC-G proteins from the genomes of *S. pombe *(Pdr1), *A. oryzae*, *P. chrysogenum *and *P. graminis*, could not be assigned to any of the groups described above. Their phylogenetic relationships remain unclear.

### Non-classified ABC proteins

**Figure 10:**
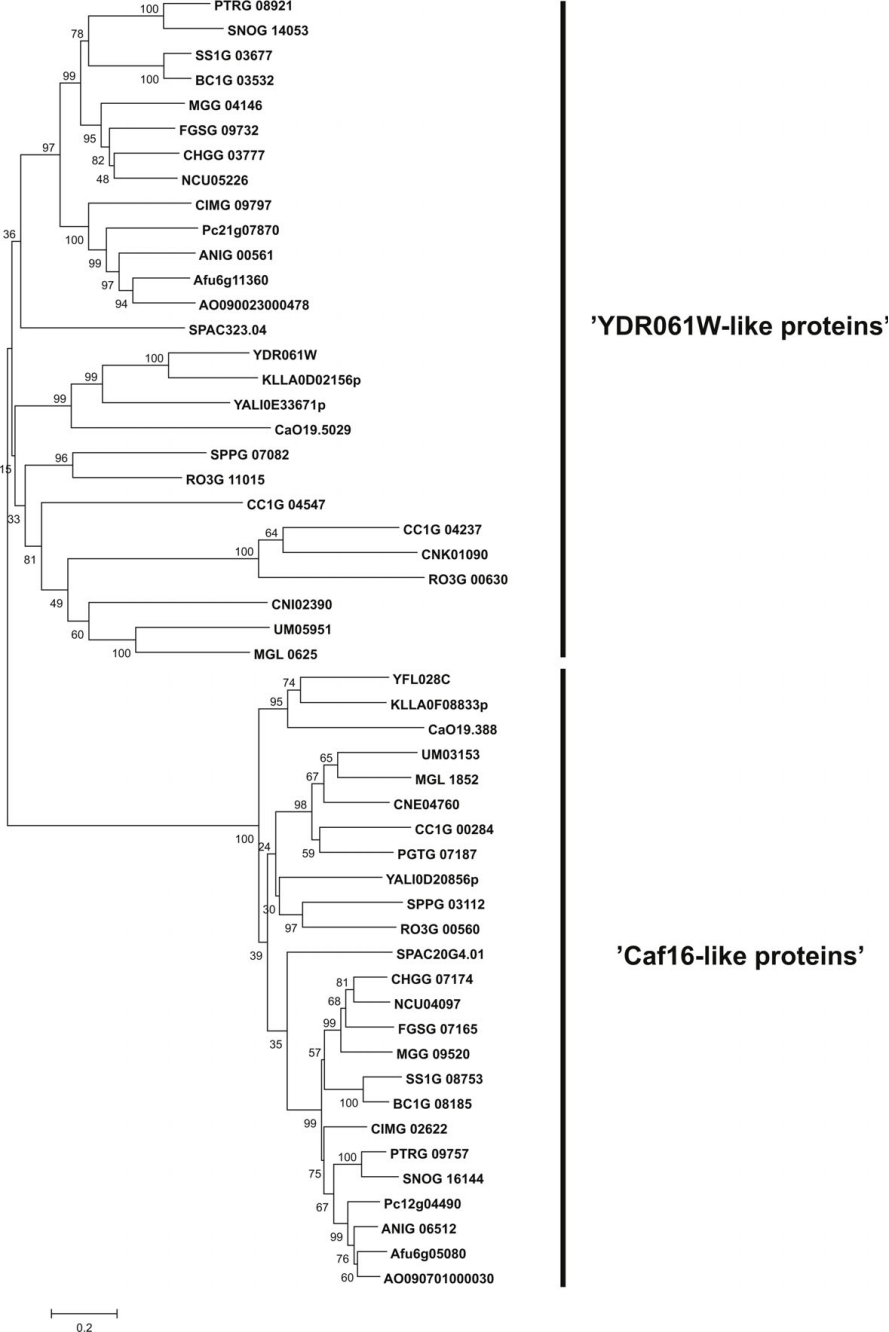
**Phylogenetic tree of unclassified fungal ABC proteins**. The tree was generated as described in the legend to the Figure 3.

In addition to the members of the well-defined subfamilies ABC-A to G, fungal genomes also contain several genes encoding ABC proteins that can not be classified into any of the known groups (Figure 10). These proteins lack transmembrane domains, but their amino acids sequences of the NBDs clearly demonstrates that they belong to the superfamily of ABC proteins. One of these groups consists of proteins related to *S. cerevisiae *Caf16p (YFL028C), and these are found in all analyzed species except for *E. cuniculi *and *B. dendrobatidis*. Caf16p is part of the CCR4-NOT transcriptional regulatory complex involved in controlling mRNA initiation, elongation and degradation [105]. However, the role for Caf16p within this complex is currently not well understood.

The prototypical member of the second group of fungal non-classified ABC proteins is *S. cerevisiae *YDR061W. It displays similarity to the components of bacterial multisubunit ABC systems. However, no corresponding transmembrane components have been found, and thus the existence of such multisubunit ABC system in fungi remains questionable. Related proteins are found in most of the species analyzed, with the exception of *E. cuniculi*, *B. dendrobatidis*, and *P. graminis*. Little is known about the function of those proteins in fungi. They are present in a single copy in most of the analyzed species, but there are two members of this branch present in *R. oryzae*, *C. cinerea*, and *C. neoformans*.

## Conclusions

Our analysis of fungal ABC proteins provides an insight into the diversity of this group of proteins within the main fungal lineages. It shows that ABC proteins are a highly dynamic group that has undergone a significant diversification after the divergence of fungal phyla (chytridiomycetes, 'zygomycetes', ascomycetes, and basidiomycetes). The process of gene duplication was apparently accompanied by gene loss events, and this resulted in a great variety of ABC proteins that we can observe today in the fungal genomes. Clearly, two evolutionary scenarios can be recognized. The number of members of the 'small' subfamilies (ABC-A, -D, -E, -F, half-size ABC-B proteins, and unclassified ABC proteins) remained low and quite constant in the analyzed species, with only few exceptions, like an amplification of the ABC-F subfamily in *R. oryzae*. On the other hand, there is a remarkable variation in the number of members of the 'large' subfamilies (ABC-B, -C, and -G) between the different species. These differences can be as high as 11-fold in the case of ABC-G proteins. The reasons for the massive proliferation of ABC-G proteins, most notably in species of the *Pezizomycotina *group, is currently not well understood. This is because of the very limited information available about the physiological functions of fungal ABC transporters in general except for *S. cerevisiae *and *S. pombe*.

Few ABC proteins are strictly required for cell viability. For example, in *S. cerevisiae *there are only three essential ABC proteins, Yef3p, Arb1p, and Rli1p, none of which is involved in transport. The number of essential ABC proteins might be larger in filamentous fungi, especially with respect to the mitochondrial transporters. On the other hand, an extensive amplification of ABC transporters in fungal genomes may have resulted in their partial functional redundancy. Trying to define a 'minimal' set of ABC proteins in fungi, we focused on two species that contain the lowest numbers, namely *E. cuniculi *and *S. pombe *with 13 and 19 ABC proteins, respectively. *E. cuniculi *is a highly specialized parasitic species with a very compact genome. Members of only four out of the 7 eukaryotic subfamilies of ABC proteins were found in this species (ABC-B, -E, -F, and-G), and ABC proteins are only distantly related to those of other fungi. So, this species can hardly serve as a good model for fungi in general. On the other hand, the set of *S. pombe *ABC proteins may be close to the minimal set needed for a free-living organism, as it is among the smallest of the sequenced eukaryotic genomes according to the TransportDB database [106]. It lacks ABC-A proteins and even more remarkably it is the only free-living species in our analysis that lacks peroxisomal ABC-D transporters, although peroxisomes are present in this organism. At the same time, there are no counterparts of two *S. pombe *transporters, Pmd1 and Hmt1, in the genomes of *Saccharomycetales*, indicating that even a further reduction of the number of ABC transporters might be possible.

Three species, *S. punctatus*, *B. dendrobatidis *and *R. oryzae*, were of special interest as they represent basal lineages that received only little attention as compared to asco- and basidiomycetes. Some of ABC proteins from these species could be assigned to the well-defined groups of orthologous proteins (like Atm1-, Mdl1-, or Hmt1-related transporters) that are highly conserved throughout the fungal kingdom. However, there are also a lot of lineage-specific proteins that form separated clusters in the phylogenetic trees. Especially remarkable is the coexistence of full-length and half-size ABC-A transporters in the genomes of chytrid fungi, which is unique among the species included in our analysis.

Sets of ABC proteins in the genomes of basidiomycetes and ascomycetes (in particular members of the subphylum *Pezizomycotina*) have a lot in common. The number of members of the particular subfamilies in the genomes of basidiomycetes, however, tends to be lower than in those of ascomycetes, and *P. graminis *has the lowest number of ABC proteins per 1 Mb of genome among analyzed species (Additional file 2). Within the phylum *Ascomycota*, there is a trend towards an increase of the number of ABC proteins. This is especially evident within the subphylum *Pezizomycotina *(especially prominent in *Aspergillus *species and in *G. zeae*), while *S. pombe *and members of the order *Saccharomycetales *contain a significantly reduced set of ABC proteins, as several of the groups of ABC-proteins present in other ascomycetes are missing from their genomes. Some distinct features of the *S. pombe *transportome were already discussed above. Members of *Saccharomycetales *also lack ABC-A proteins, with a notable exception of *Y. lipolytica *indicating that these systems were most likely lost after the divergence of the lineage leading to this species from the rest of the group. The full-length ABC-B transporters in *Saccharomycetales *are represented by a single protein, the mating pheromone transporter Ste6p/Hst6 (again, *Y. lipolytica *is an exception from this rule, as it has 3 additional full-length ABC proteins). Finally, *Saccharomycetales *species (once again, except for *Y. lipolytica*) are characterized by the duplication of the gene encoding the mitochondrial half-size transporters Mdl1. Higher ascomycetes belonging to the subphylum *Pezizomycotina *contain the most diverse sets of ABC proteins, with several groups of proteins specific for this subphylum. Unfortunately, the information about their physiological functions is still scarce and mainly restricted to multidrug resistance.

The expansion of ABC proteins must have occurred frequently and independently in different lineages of the fungal kingdom. The difficulty in establishing groups of orthologous proteins poses problems for the nomenclature. So far, the only fungal species with names assigned to all ABC proteins are *S. cerevisiae *and *S. pombe*. However, those gene names can hardly be used to classify the diversity of fungal ABC proteins simply because the two model species lack many of the ABC proteins found in filamentous fungi. Therefore, we suggest to adopt a nomenclature system recently proposed for plant ABC proteins [12]. According to this system, the name of a given fungal ABC transporter should contain a species identifier based on a Latin binomial (e.g., Sc for *S. cerevisae*), the subfamily abbreviation (e.g. ABCB) and a number for each gene family member (e.g. ABCB1, ABCB2 and so forth). Use of such nomenclature should help to cope with an increasing amount of data about fungal ABC proteins produced by numerous genome sequencing projects.

Another problem is the lack of functional data on fungal ABC transporters. Most of the available information was obtained from studies on *S. cerevisiae *and *S. pombe*. Both species have significantly reduced set of ABC proteins and cannot serve as a good model for other fungi. Only a small fraction of ABC proteins found in the remaining species have been characterized so far, but for the majority the function is unknown. In fact, most of the papers published on fungal ABC transporters in the last decade deal with the role of these transporters in the multidrug resistance, leaving other aspects of their biology unnoticed. This view is, however, slowly changing as it has become increasingly clear that the physiological roles of the proteins previously described as multidrug transporters is much broader. Our analysis contributes to the functional characterization of the fungal ABC proteins in several ways. First, it identified orthologues of the well-characterized ABC proteins thus providing a first clue about their function. It also highlights the areas of special interest and that have only been marginally studied thus far. One of those is a role of ABC-A proteins in fungi. This group appears to be much more abundant within the fungal kingdom than previously realized. Its function(s), however, remains less understood, in part because of the absence of members from the genomes of both *S. cerevisiae *and *S. pombe*. Another largely unexplored area concerns the role of fungal ABC proteins in secondary metabolism. It is well known that ABC transporters are often found within prokaryotic secondary metabolism clusters where they contribute to the excretion of final products. Our analysis shows that some of the transporters associated with secondary metabolism clusters are highly conserved within fungi. Further experiments are needed to uncover their role within such clusters.

Although our analysis does not cover the whole set of ABC proteins present in the databases, it provides an insight into their astonishing diversity. The obtained results can be used as a starting point for the development of an universal classification system of fungal ABC proteins and for a further in-depth characterization of members of this highly important group.

## Methods

To identify gene loci encoding ABC proteins in the fungal genomes, multiple blastp and tblastn searches against selected genomes were performed either at the National Center for Biotechnology Information website or at the website of the Broad Institute. Sequences of *S. cerevisiae *and *A. nidulans *ABC proteins representing all known subfamilies were used as queries, so that two searches per subfamily (except for ABC-A, for which no *S. cerevisiae *members are known) were performed. All hits producing E-values below 10^-4 ^were further analyzed. For the genome sequences deposited at the Broad Institute, produced data sets were additionally checked against the list of sequences containing PFAM protein domains 'ABC transporter', 'ABC-2 type transporter', and 'ABC transporter transmembrane region'.

Phylogenetic analysis was performed with the program package MEGA4 [107] using neighbor-joining, minimum evolution and maximum parsimony algorithms and bootstrapping with 500 replicates. First, ABC proteins of each species were separated into subfamilies by their comparison with *S. cerevisiae*, *A. nidulans*, *Homo sapiens*, *Arabidopsis thaliana*, and *Dictyostelium discoideum *transporters. Afterwards, members of each subfamily and, in the case of ABC-B transporters, also full-length and half-size proteins were analyzed separately. Although the topologies of the phylogenetic trees produced with the different algorithms showed some minor differences, the same major groups of ABC proteins were recognized with all three algorithms.

## Authors' contributions

AK performed an identification and phylogenetic analysis of ABC proteins from the selected fungal genomes and wrote the manuscript. AJMD initiated the study, participated in its coordination and helped to draft the manuscript. Both authors read and approved the final manuscript.

## Supplementary Material

Additional file 1 **Table S1**. Accession numbers, loci, and amino acid sequences of all ABC proteins used in the phylogenetic analysis. Click here for file

Additional file 2 **Table S2**. Distribution of the subfamilies of ABC proteins among analyzed fungal species. Numbers given in parentheses include putative pseudogenes. Click here for file

Additional file 3 **Table S3**. Distribution of the phylogenetic groups of ABC proteins identified in our analysis among analyzed species. Numbers given in parentheses include putative pseudogenes. Click here for file

Additional file 4 **Figure S1**. Phylogenetic tree of fungal full-length ABC-B proteins (traditional view). Click here for file

Additional file 5 **Figure S2**. Phylogenetic tree of fungal half-size ABC-B proteins (traditional view). Click here for file

Additional file 6 **Figure S3**. Phylogenetic tree of fungal ABC-C proteins (traditional view). Click here for file

Additional file 7 **Figure S4**. Phylogenetic tree of fungal ABC-D proteins (traditional view). Click here for file

Additional file 8 **Figure S5**. Phylogenetic tree of fungal ABC-E and ABC-F proteins (traditional view). Click here for file

Additional file 9 **Figure S6**. Phylogenetic tree of fungal ABC-G proteins (traditional view). Click here for file
