# Supplementary material for: Phylogenetic analysis of fungal ABC transporters
Source: BMC Genomics. 2010 Mar 16;11:177. doi: 10.1186/1471-2164-11-177 (PMC2848647; doi:10.1186/1471-2164-11-177)
Supplement: Additional file 2 — Table S2. Distribution of the subfamilies of ABC proteins among analyzed fungal species. Numbers given in parentheses include putative pseudogenes. [file 1471-2164-11-177-S2.DOC]

| **Species** | **ABC-A** | **ABC-B** | | **ABC-C** | **ABC-D** | **ABC-E** | **ABC-F** | **ABC-G** | **others** | **total** | **genome size** | **transporters per Mb of genome** |
| --- | --- | --- | --- | --- | --- | --- | --- | --- | --- | --- | --- | --- |
|  |  | **full** | **half** |  |  |  |  |  |  |  |  |  |
| ***Encephalitozoon cuniculi* GB-M1** | 0 | 0 | 6 | 0 | 0 | 1 | 1 | 5 | 0 | **13** | 2.5 | 5.2 |
| ***Batrachochytrium dendrobatidis* JEL423** | 5(13) | 2(3) | 3 | 17(18) | 2 | 1 | 5 | 6 | 0 | **41(51)** | 23.7 | 2.15 |
| ***Spizellomyces punctatus* DAOM BR117** | 4 | 6(7) | 3 | 11(12) | 3 | 1 | 6 | 11(13) | 2 | **47(51)** | 24.13 | 2.11 |
| ***Rhizopus oryzae* RA99-880** | 0 | 4 | 5 | 7 | 4(5) | 2 | 10 | 10 | 3 | **45(46)** | 40 | 1.15 |
| ***Schizosaccharomyces pombe* 972h-** | 0 | 2 | 3 | 4 | 0 | 1 | 5 | 2 | 2 | **19** | 14 | 1.36 |
| ***Saccharomyces cerevisiae* S288c** | 0 | 1 | 3 | 6 | 2 | 1 | 5 | 10 | 2 | **30** | 12.07 | 2.49 |
| ***Kluyveromyces lactis* NRRL Y-1140** | 0 | 1 | 3 | 4 | 2 | 1 | 4 | 6 | 2 | **23** | 10.69 | 2.15 |
| ***Candida albicans* SC5314** | 0 | 1 | 3 | 4 | 2 | 1 | 4 | 9 | 2 | **26** | 16 | 1.63 |
| ***Yarrowia lipolytica* CLIB122** | 1 | 4 | 2 | 10 | 2 | 1 | 4 | 6 | 2 | **32** | 20.5 | 1.56 |
| ***Aspergillus fumigatus* Af293** | 1 | 10(12) | 3 | 11(12) | 2 | 1 | 5(6) | 15 | 2 | **50(54)** | 30 | 1.8 |
| ***Aspergillus nidulans* FGSC A4** | 1 | 7(9) | 3 | 11(13) | 2 | 1 | 5 | 16 | 2 | **48(52)** | 31 | 1.68 |
| ***Aspergillus oryzae* RIB40** | 1 | 14 | 5 | 18(23) | 2 | 1 | 5 | 22 | 2 | **70(75)** | 37 | 2.03 |
| ***Penicillium chrysogenum* Wisconsin 54-1255** | 0 | 10 | 5 | 9(10) | 2 | 1 | 5 | 19(20) | 2 | **53(55)** | 32 | 1.72 |
| ***Coccidioides immitis* RS** | 1 | 6 | 5(9) | 5(6) | 2 | 1 | 4 | 8 | 2 | **34(39)** | 28.89 | 1.35 |
| ***Pyrenophora tritici-repentis* Pt-1C-BFP** | 1 | 8 | 4 | 6(7) | 2 | 1 | 5 | 11 | 2 | **40(41)** | 37.8 | 1.08 |
| ***Phaeosphaeria nodorum* (*Stagonospora nodorum*) SN15** | 2 | 7(9) | 5 | 9 | 2 | 1 | 5 | 10 | 2 | **43(45)** | 37.24 | 1.21 |
| ***Sclerotinia sclerotiorum* 1980** | 1 | 7 | 3(4) | 11(12) | 2 | 1 | 5 | 11(13) | 2 | **43(47)** | 38 | 1.24 |
| ***Botryotinia fuckeliana* (*Botrytis cinerea*) B05.10** | 2 | 8(9) | 4 | 14(15) | 2 | 1 | 5 | 12(13) | 2 | **50(53)** | 42.66 | 1.24 |
| ***Magnaporthe grisea* 70-15** | 2 | 12 | 6(7) | 10(11) | 2 | 1 | 5 | 8 | 2 | **48(50)** | 40 | 1.25 |
| ***Gibberella zeae (Fusarium graminearum)* PH-1** | 1 | 10 | 6 | 16 | 2 | 1 | 5 | 19 | 2 | **62** | 36.45 | 1,7 |
| ***Chaetomium globosum* CBS 148.51** | 1 | 7 | 5 | 8(9) | 2 | 1 | 5 | 9 | 2 | **40(41)** | 34.89 | 1.75 |
| ***Neurospora crassa* OR74A** | 1 | 5 | 4 | 9 | 2 | 1 | 4 | 7 | 2 | **35** | 43 | 0.81 |
| ***Coprinopsis cinerea (Coprinus cinereus)* okayama7#130** | 1 | 5(7) | 9 | 12 | 2 | 1 | 5 | 7 | 3 | **45(47)** | 37.5 | 1.25 |
| ***Cryptococcus neoformans var. neoformans* JEC21** | 0 | 2 | 4 | 9 | 2 | 1 | 4 | 8 | 3 | **33** | 19.05 | 1.73 |
| ***Ustilago maydis* 521** | 1 | 5 | 3 | 10(12) | 2 | 1 | 5 | 8 | 2 | **37(39)** | 20.5 | 1.9 |
| ***Malassezia globosa CBS7966*** | 0 | 4 | 3 | 5 | 2 | 1 | 4 | 2 | 2 | **23** | 9 | 2.56 |
| ***Puccinia graminis* f. sp. *tritici* CRL 75-36-700-3** | 0 | 1 | 3 | 7 | 2 | 1 | 4(5) | 7 | 1 | **26(27)** | 81.5 | 0.33 |
